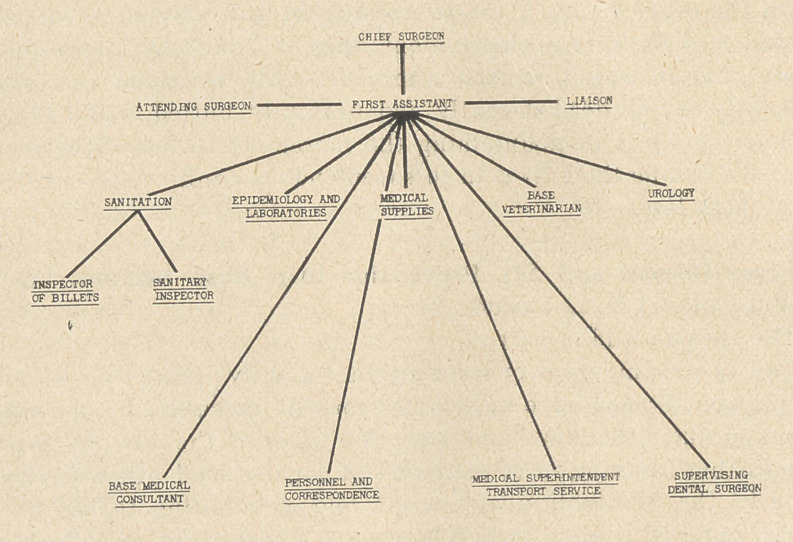# Methods of Feces Disposal and Fly Prevention in a Base Section

**Published:** 1918-10

**Authors:** H. C. Coburn


**Feces Dispoal and Fly Prevention in a Base Section**. Lieutenant-Colonel H. C. COBURN, M. C.

The speaker said in part :

The various methods of feces disposal and fly prevention in use throughout France have been so thoroughly discussed by the previous speakers that I will not take the time of the Society with any further description of the types of latrines and other devices used in the elimination of excreta and combatting the fly problem, but will confine myself to a short summary of the administrative side of the question from the point of view of a Base Surgeon's office.

A discussion of feces disposal and fly prevention in a base section may be considered to involve practically any part of France except the front. In fact the base with which I am most familiar covers about one sixth of all France, with a terrain varying from many miles of sea coast with adjacent low lying sandy soil and ground water within two or three feet of the surface, through pine covered plains, back to hilly and finally mountainous districts with some of the highest peaks found in this part of Europe. It is thus evident that no one scheme of disposal can be applied to all parts of such an area. Excreta, for example, may in some districts be conveyed to water courses by means of modern sewerage systems; in other sections the pit or tinette type of latrine may be used ; the method depends upon location and the availability of supplies.

The activities of a base section may be many and varied. Along the estuaries on coast are docks for the reception of troops and freight, adjoining which we find colonies of stevedores, labor troops, civilian construction companies, Algerians and Annamites, all living in close proximity in necessarily contracted camp sites. These are usually surrounded by civilians, crowded as near as possible to the camps, either by accident or for purposes of trade. Of course the sanitation of these camps is a matter of grave concern and constant vigilance. Rest camps for the temporary housing of incoming troops, organization and training centers where their education is perfected, and large training camps for the same purpose, holding as high as three brigades each with seven to eight thousand horses, are found near the ports. Stretching back from the sea through sandy plains covered with pine woods are numerous forestry camps. These have been noted among the natives for the large number of flies found there every summer, possibly due to the heat retaining properties of the sand facilitating the hatching of larvae, granted the presence of the necessary organic matter. Besides the camps, troops are billeted throughout the bases in villages with the ever present manure pile, the home-made latrine and their mutual friend the ubiquitous fly. Base and camp hospitals and infirmaries, prison camps, remount depots and veterinary hospitals are among the various activities, each of which has its own individual problems and methods of solution.

Organization : The supervision of sanitation in such extensive areas, with their divergent enterprises, necéssitates a well considered plan of co-operation, by means of which the care of individual units may be correlated and maintained under the direction of a central head. This resolves itself into the question of local and central administrative control.

Local control : The commanding officer of each camp or individual formation is responsible for the sanitary condition of the organizations under his command. Through the line officer detailed as " police officer " the necessary sanitary details for clean up work are handled. Daily inspections of the various camp areas are made by the regimental surgeon who reports to the regimental commander any irregularities found. Each permanent camp has a Camp Surgeon, a senior medical officer, who remains on duty there irrespective of the shifting of the organizations temporarily in camp, and who has supervision of the medical work of the camp and general charge of the sanitary situation. Under his direction is the Camp Sanitary Inspector, an officer of the Medical or Sanitary Corps, whose duty it is to make a thorough daily inspection of the camp and report, through the Camp Surgeon to the permanent Camp Commander, any breaches of sanitation that cannot be rectified locally.

In the base where I am on duty the Chief Surgeon has been instrumental in obtaining an order from the Commanding General of the section turning over to each camp police officer for sanitary work each day details from line troops according to the following schedule :

| | | |
|---|---|---|
| up to 1000 | 2 o/o | of entire command |
| 1000 to 5000 | 1/2 1/2 o/o | " " " |
| 5000 to 1000 | 1 o/o | " " " |
| 10000 and over | 1/2 o/o | " " " |

For all other troops and detachments 2 men per organization (G. O., 41., Hq. B. S. 2).

These men perform the duties of sanitary squads. The system has worked out most satisfactorily. It must be said that the co-operation of line officers in sanitary work, from commanding generals down to junior subaltern, has been excellent. Our old officers of the former regular army have through years of experience seen the necessity for the observance of sanitary rules, and officers of less practical knowledge, by the courses on field and camp sanitation and care of troops, have been taught the need of maintaining their men in good physical condition. As a rule, poor camp sanitation, with its sequella of communicable disease, is an indication of ignorance or lack of attention to sanitary principles on the part of the medical officer rather than officers of the line. The strong or weak link in the sanitary chain is the regimental or camp surgeon. Our medical schools at home have turned out good physicians and surgeons capable of good performance when called into military service, provided they are assigned to hospital work. In the ordinary medical school, however, little attention is paid to the subject of hygiene and sanitation. The average student regards the subject as a useless bore, and usually passes the examination by a night's cramming, proceeding to forget it as soon as possible thereafter. Military sanitation is usually not touched upon at all, in consequence of which most medical officers come to the army untrained in this most important subject, and entirely unfamiliar with its principles. With the present raw and comparatively undisciplined state of our troops, fresh from concentration camps, much of our work of organization, and I may say its most important part consists in the education of medical officers assigned to duty with the various combatant or labor organizations.

Central control : The office organization, elaborated by the Chief Surgeon of Base Section N° 2., is illustrated in the accompanying diagram. It is an old army axiom that the success or failure of a command rests with the man at the top whose skill in administration and in the selection of his assistants determines the future of his project. In this case the Chief Surgeon is responsible for ideas, subordinates for carrying them out. Each assistant is responsible for the work in his department and it is handled by him so far as he is able, unless some advice on the part of the Chief Surgeon is required. Information covering the activities of the various sub-departments is obtained by means of conferences several times a week, at which each officer gives a brief summary of the current work of his department, and the ideas of the various assistants are thus correlated and exchanged.

Education : Various orders, bulletins, circulars and memoranda prepared in the Chief Surgeon's office, usually by the Director of the Base Laboratory or his assistant the Base Epidemiologist, are published from time to time for the information of medical officers throughout the base. These usually take the form of various health bulletins, or may follow the plan of circulars from the Office of the Chief Surgeon, A. E. F. They aim to call attention in advance to diseases which may be expected to prevail during various seasons, and through them an effort is made not only to acquaint officers with epidemiological conditions prevailing throughout the section but actually to educate them in the etiology, diagnosis and treatment of various preventable illnesses; for instance, bulletins have been furnished on the subjects of dysentery in its various forms, Vincent's angina, angina, fever of undertermined origin (three day fever), and one on pneumonia is now in process of preparation. These are all of considerable interest and value to medical officers, situated at a distance from headquarters, in keeping them informed of diseases to be expected throughout their area. Where the seriousness of the situation warrants it the Commanding General publishes certain of these in the form of a general order, thus lending to the recommendation the force of military authority. Along similar lines is the issue of blue prints and diagrams of various forms of sanitary apparatus and appliances, including pit and tinette type latrines, grease traps, dumps, food cabinets and cold storage boxes. These are forwarded to all camps and are simple of construction and accompanied by sufficient directions to make their establishment easy.

In line with the system of office conferences, mentioned above, are our monthly meetings of medical officers within access of the section headquarters. These are planned with the idea of acquainting officers on duty in camps or with troops of the sanitary needs and service of the base, with special reference to prevailing preventable disease. At these meetings pertinent medical topics are discussed and they have a definite value in augmenting the spirit of co-operation among medical officers, and establishing among them a feeling that the Chief Surgeon's Office is doing its share in aiding them in their work.

Through the co-operation of the commanding officer and senior officers on duty at one of the base hospitals near our headquarters, a post graduate medical school has been established for the benefit of any medical officers who may be in rest camps or temporarily attached to units in the neighborhood awaiting definite assignment elsewhere. As this particular hospital is one of the largest in our army, there is always an opportunity for such officers to be temporarily assigned there and for them to work as dressers or assistants in wards. A course of lectures, demonstrations, and bedside teachings have been organized by Major Richard C. Cabbot and his assistants, by means of which these officers derive exceedingly valuable instruction from some of our best American teachers. Meanwhile they are given lectures and demonstrations on sanitary topics and are taken at intervals to the various camps in the neighborhood and the good and bad points of sanitation there existing are shown them. This is really one of the most satisfactory branches of our work and I have yet to see a medical officer who attended this little school who did not speak in the highest praise of the efforts shown there to assist him in his work in France.

The question of inspection will be considered later. It may be noted here, however, that one of the most important functions of all inspecting officers is to give the maximum amount of information and advice rather than useless criticism. It is most satisfying for an inspector to be told upon his return to an outlying station that his advice has resulted in local improvement.

Billets : When billets are being selected for troops about to arrive, a medical officer accompanies the billeting officer on his round through the various villages. Billets are selected with due consideration for sanitary needs and the proper attention is paid to medical opinion. Arrangement has been made by which the proper sanitary appliances are kept on hand by the Base Quartermaster and placed in billeting areas in advance of the arrival of troops. Latrine boxes, crude oil and sprayers, garbage and latrine cans, fly traps, burlap and lumber are supplied in sufficient quantities according to a schedule worked out on a percentage basis, with reference to the number of troops due to occupy the village or town. Incoming troops, therefore, find no excuse upon arrival for sanitary derelictions. They are met or seen shortly after arrival by the Medical Inspector of Billets who furnishes information on sanitary subjects, gives advice as to local problems, informs medical officers relative to procedure for obtaining medical supplies and leaves copies of a sanitary memorandum covering the subjects of latrines, urine cans, garbage and refuse disposal, and the care of kitchens and incinerators.

Inspections : Routine monthly inspections covering the medical and sanitary features of all activities in the base are made regularly by the Base Sanitary Inspector. At these visits he notes particularly the disposal of wastes, cleanliness and care of kitchens, mess halls and garbage receptacles, construction of latrines, methods of urine disposal, manure dumps, incinerators and fly prevention and extermination. He renders an informal report to the Chief Surgeon covering each camp inspected; in addition one of his main functions is to teach the more inexperienced medical officers the general principles of camp and field sanitation, and to take up with them their local problems and assist in their solution. The Base Medical Consultant makes similar monthly rounds, preferably at the same time as those of the Sanitary Inspector. While the Sanitary Inspector is investigating the details covering preventive medicine, the Base Medical Consultant goes through the hospitals or infirmary wards, checks up the diagnosis and treatment of cases and obtains valuable information relative to the frequency of certain phases of disease which may have occurred not only among patients admitted to hospital but among those applying for treatment at sick-call and not marked sick upon the sick records. If any data of epidemiological interest is found, it is transmitted to the Base Epidemiologist upon the conclusion of his inspection trip. The Medical Inspector of Billets makes weekly inspection of the troop areas under his care. He acts as special advisor to all the medical officers stationed with troops in villages. Usually these troops are among the latest arrivals in the base and know less how to care for themselves than the men who have passed through this period and have become established in training camps or centers. For that reason the Medical Inspector of Billets should be a man of considerable experience in field work and possessed of administrative ability, intelligence, and tact. His position is one of the most important of the administrative force since his clientele are apt to be untrained and inexperienced and changing from week to week.

Frequent inspections are made by the Chief Surgeon or his assistant to keep themselves personally informed of conditions in general throughout the section and for the purpose of seeing medical officers at work. The successful administrator should keep his assistants as busy as possible, leaving himself free for the greater part of each day to plan the solution of new problems. If he can visit one camp or billet a day in addition, his time is counted well spent.

Special inspections may be called for on account of reports of sanitary delinquencies or the presence of preventable disease. The Sanitary Inspector is called on frequently to make such trips. However rigid routine inspections may be, or however carefully the Chief Surgeon may supervise his base, there will be conditions reported from time to time which necessitate an immediate visit from some qualified medical officer. This takes up a large part of the Sanitary Inspector's time. In our base section it is estimated that the Sanitary Inspector can make the necessary trip to each formation now existing in the base, exclusive of billeting areas, once each month, and can cover the entire situation in a period of three weeks, this by means of constant travelling and allowing no time for him to digest the results of his trips and make his reports, except by working at night. The remaining week would be fully occupied by the examination of the routine monthly sanitary reports submitted to the Chief Surgeon's office each month, from all organizations in the area, in addition to which he may be called upon for special inspections. The office personnel should be of sufficient size to count upon a regular visit each month, as above, in which case an assistant would probably be necessary to aid in this work and the special inspection trips. In the presence of epidemics or threatened epidemics, the Base Epidemiologist or his assistant, usually with the Director of the Base Laboratory, proceeds at once to the spot. An additional medical officer trained in laboratory work, with a portable field laboratory outfit, is often called into service. The Base Medical Consultant is sometimes called upon for similar work, but his particular role is as consultant in the diagnosis and care of medical cases. He is not so apt to be called upon to make inspection trips of this character unless unusual medical conditions present themselves or there is some question as to the proper care of the sick.

While it is realized that these remarks have perhaps diverged from the actual question of feces disposal and fly prevention in a base section, it is hoped that they may have been of some value by giving an insight into the administrative difficulties involved in caring for a large base and showing what steps are being taken to hold the situation in hand. To summarize, it may be said that success depends upon :

First : An efficient and well thought out central organization with definite duties assigned to each assistant, and the correlation of individual ideas and experience by means of frequent conferences.

Second : Education of relatively untrained medical officers.

Third : Advance preparation of camps and billets and the initiation of proper efforts to give newly arrived troops a good start.

Fourth : Frequent inspections, to prevent the formation, and effect the correction, of insanitary habits.

## Figures and Tables

**Figure f1:**